# Monitoring of manufacturing process using bayesian EWMA control chart under ranked based sampling designs

**DOI:** 10.1038/s41598-023-45553-x

**Published:** 2023-10-25

**Authors:** Imad Khan, Muhammad Noor-ul-Amin, Dost Muhammad Khan, Emad A. A. Ismail, Wojciech Sumelka

**Affiliations:** 1 Department of Statistics, Abdul Wali Khan University Mardan, Mardan, Pakistan; 2 COMSATS University Islamabad, Lahore Campus, Lahore, Pakistan; 3 Department of Quantitative Analysis, College of Business Administration, King Saud University, Riyadh, 11587 P.O. Box 71115, Saudi Arabia; 4 Institute of Structural Analysis, Poznan University of Technology, 60-965 Poznan, Poland

**Keywords:** Mathematics and computing, Applied mathematics, Statistics

## Abstract

Control charts, including exponentially moving average (EWMA) , are valuable for efficiently detecting small to moderate shifts. This study introduces a Bayesian EWMA control chart that employs ranked set sampling (RSS) with known prior information and two distinct loss functions (LFs), the Square Error Loss function (SELF) and the Linex Loss function (LLF), for posterior and posterior predictive distributions. The chart's performance is assessed using average run length (ARL) and standard deviation of run length (SDRL) profiles, and it is compared to the Bayesian EWMA control chart based on simple random sampling (SRS). The results indicate that the proposed control chart detects small to moderate shifts more effectively. The application in semiconductor manufacturing provides concrete evidence that the Bayesian EWMA control chart, when implemented with RSS schemes, demonstrates a higher degree of sensitivity in detecting deviations from normal process behavior. Comparison to the Bayesian EWMA control chart using SRS, it exhibits a superior ability to identify and flag instances where the manufacturing process is going out of control. This heightened sensitivity is critical for promptly addressing and rectifying issues, which ultimately contributes to improved quality control in semiconductor production.

## Introduction

In manufacturing industries, it is common for processes to undergo variations that cannot be entirely eliminated naturally. To address this inherent challenge, control charts (CCs) are indispensable statistical tools for monitoring processes and effectively mitigating abrupt deviations from the desired target. As a result, CCs play a pivotal role in significantly reducing the likelihood of producing defective items. In the context of the production process, two distinct types of variations exist common cause and special cause. When a process exhibits common cause variation, it is perceived as being under control. Conversely, if the variation arises from a special cause, the process is deemed to be out-of-control. Shewhart^1^ introduced CC techniques that rely solely on current sample information to monitor the production process. These techniques have demonstrated high effectiveness in detecting significant shifts in process parameters. Furthermore, Page^2^ and Roberts^3^ have introduced alternative CCs, such as the Cumulative Sum (CUSUM) and EWMA CCs, respectively. These charts present distinct methods for monitoring and identifying deviations in a process, offering opportunities to enhance process control and ensure product quality. These specialized charts are specifically designed to improve sensitivity in detecting smaller or moderate shifts within the production process. Numerous researchers have made significant contributions to the advancement of memory-type CCs. Among them are Lucas and Crosier^4^, Khoo^5^, Khan et al.^6^, and Saghir et al.^7^. Abbas et al.^8^ examined CCs monitoring processes, with a focus on both location and dispersion. We investigated dispersion CCs using RSS, MRSS, and ERSS, and found that they outperform traditional methods, leading to improved process quality and efficiency. Abbas et al.^9^ present a dispersion control chart that utilizes NRSS, enhancing precision and resilience across diverse domains such as quality control, environmental monitoring, and process control. NRSS incorporates expert knowledge and sample selection to reduce the impact of outliers and accommodate non-normal data, with validation using real-world chemical reactor data. In Mohammadkhani et al.^10^ study, they explore the efficiency of RSS in statistical process monitoring and CC design, emphasizing its cost-effectiveness in obtaining representative samples. They also review the literature to identify research gaps and provide recommendations for future studies. Noor-ul-Amin and Tayyab^11^ enhanced the EWMA CC using cost-effective RSS methods, such as PDRSS, EPDRSS, and QPDRSS, finding that these charts outperform the SRS-based classical EWMA CC in detecting small to moderate process mean shifts, supported by an illustrative application.

Statistical inference is a fundamental process comprising two primary components: estimation and hypothesis testing. Estimation involves determining unknown population parameters, and two main approaches are used: classical and Bayesian. In the classical approach, estimators rely on data or sample information without considering prior knowledge or beliefs about the population parameter. Essentially, classical estimation relies solely on observed data to make inferences about the parameter of interest, without incorporating additional contextual information or prior understanding. In the Bayesian approach, estimators incorporate both the available sample data and prior knowledge or beliefs about the population parameter. For instance, Menzefricke^12^ introduced a CC within the Bayesian framework to monitor the unknown location parameter and also explored CCs for monitoring variance^13^. In another study, Menzefricke^13^ extended the use of the EWMA CC within Bayesian theory. They applied a normal distribution to model the population variance and assessed the Bayesian EWMA CC effectiveness in monitoring both the process mean and variance. The Bayesian CC construction involved leveraging the posterior (P) distribution to make informed inferences about the underlying parameter. This Bayesian approach allows for a more comprehensive and contextually informed estimation of process parameters, improving the monitoring and control of industrial processes. In the Bayesian studies, the LF plays a crucial role in minimizing the risk associated with the estimator. Tsui and Woodall^14^ conducted a study on multivariate CCs, assessing their effectiveness across different LFs. Wu and Tian^15^ introduced a CUSUM CC capable of detecting variations in both process mean and variance by utilizing a weighted LF Serel^16^ study delves into the economic design of EWMA-based CCs employing various LFs. Computational methods are employed to ascertain optimal chart parameters, underscoring the significance of adapting the sampling interval over sample size or control limits in response to shifts in the cost of poor quality. This observation is supported by numerical comparisons with Shewhart and S charts. Riaz et al.^17^ introduced a Bayesian EWMA approach to monitor process mean, utilizing three distinct LFs. their study incorporated informative conjugate and non-informative priors for both P and PP distributions. This Bayesian EWMA approach provides a robust framework for monitoring the process mean by considering various LFs and prior information. Noor et al.^18^ conducted research within the Bayesian framework, employing two different LFs to develop a hybrid EWMA CC for industrial process monitoring. Their study aimed to assess the performance of this chart using key metrics such as ARL and SDRL. They considered both informative and non-informative priors, demonstrating the flexibility of the Bayesian approach in adapting to different scenarios. Extensive simulations and a real-world example showcased the practicality and effectiveness of the proposed chart in real industrial applications. Asalam et al.^19^ introduced a novel Bayesian Modified-EWMA chart to monitor location parameters in processes and enhance statistical process control. Four different LFs and a conjugate prior distribution are employed. Performance evaluation using ARL demonstrates the proposed chart's superior performance in detecting small to moderate process shifts compared to existing methods. Real-life examples in the mechanical and sports industries further validate its significance. Noor et al.^20^ propose a new AEWMA CC, considering different LFs and employing conjugate priors with squared error and linex LFs. Performance assessment involves ARL and SDRL, using Monte Carlo simulations and a real-data example to compare with the existing Bayesian CC. Noor et al.^21^ investigated Bayesian EWMA CCs for non-normal lifetime distributions, including Exponential, Inverse Rayleigh, and Weibull distributions, with uniform priors and different LFs. Performance assessment based on ARL and SDRL favored the Weibull distribution as the most effective among the options. A practical example demonstrates the implementation of these charts. Liu et al.^22^ introduce a novel Bayesian AEWMA CC with multiple LFs and PRSS schemes for detecting small to moderate process mean shifts. Monte Carlo simulations and semiconductor manufacturing applications confirm its superior performance in process monitoring. Khan et al.^23^ introduce an innovative Bayesian AEWMA CC utilizing RSS designs, and an informative prior for effectively monitoring mean shifts in normally distributed processes. Extensive Monte Carlo simulations demonstrate its increased sensitivity when compared to the current exiting CC. Validation in real-world semiconductor fabrication confirms its effectiveness in detecting out-of-control signals.

The various aforementioned studies for monitoring the process mean under the Bayesian approach are done by using a SRS scheme. RSS designs is a cost-effective data collection method often applied in quality control contexts. RSS involves ranking units within groups and selecting a subset of these ranked units for sampling. Utilizing RSS can lead to improved CC efficiency and accuracy. By combining the EWMA CC with the Bayesian approach within the framework of RSS, it's possible to further enhance CC performance. This approach capitalizes on the efficient sampling technique offered by RSS and the adaptability of Bayesian updating, allowing control limits to be adjusted based on available data. The combination of RSS and Bayesian methods in EWMA CC proves particularly advantageous when data collection costs are high or sample sizes are limited. Therefore, we present a Bayesian CC that integrates three distinct RSS strategies: RSS, median RSS (MRSS), and extreme RSS (ERSS). These sampling methodologies are applied to both P and PP distributions, accommodating informative and non-informative priors. The proposed Bayesian CC incorporates two distinct LFs i.e., SELF and LLF. To assess the performance of this proposed CC across various ranked-based sampling approaches, we employ metrics such as ARL and SDRL. These performance measures are calculated using the Monte Carlo simulation method. The article structure is organized as follows: “Bayesian approach” provides an overview of Bayesian theory, encompassing essential terminology related to various LFs. “Ranked set sampling” discusses the RSS, MRSS, and ERSS schemes. The design of the proposed Bayesian EWMA CC is elaborated in “EWMA control-chart”. In “Simulation study”, we conduct a comprehensive simulation study. “Results, discussion and findings” includes a discussion of our findings, while “Real data application” presents an illustrative example. “Conclusion” and “Limitations of the study” outlines outline our conclusions and the study's limitations, and finally, in “Future recommendations”, we provide our recommendations.

## Bayesian approach

This section presents a concise discussion of the Bayesian approach, which incorporates both sample knowledge and prior information through the P and PP distribution. The prior distribution is a pivotal element in Bayesian estimation and can be divided into two primary categories: informative and non-informative priors. Informative priors come into play when there exists prior knowledge or information regarding the population parameter, enabling the integration of this prior information into the Bayesian analysis. Conversely, non-informative priors, sometimes referred to as vague or uninformative priors, are selected when limited to no prior knowledge is accessible. This choice ensures that the prior distribution does not introduce any undue influence or bias into the Bayesian inference process. Conjugate prior distributions are employed when both the informative prior and the sampling distribution belong to the same family of probability distributions. This choice streamlines Bayesian analysis by simplifying the calculation of the P distribution. The advantage lies in the P distribution maintaining the same mathematical form as the prior distribution, making computations more straightforward. In essence, selecting a conjugate prior ensures mathematical compatibility between the prior and P distributions, facilitating efficient Bayesian estimation and belief updating when new data is introduced. In the present study, the variable under consideration *X* is used which follows a normal distribution with unknown mean $\theta$ and known variance $\delta^{2}$. We considered normal prior (conjugate prior) with hyperparameters $\theta_{0}$ and $\delta_{0}^{2}$ given as:

1 $$p\left( \theta \right) = \frac{1}{{\sqrt {2\pi \delta_{0}^{2} } }}\exp \left\{ { - \frac{1}{{2\delta_{0}^{2} }}\left( {\theta - \theta_{0} } \right)^{2} } \right\}$$

when no information is available about the prior distribution $\theta$, it has minimum effect on the P distribution. Thus, in cases where no prior knowledge is available, it is often preferred to utilize a uniform distribution as the prior. This choice of using a non-informative prior, often referred to as a vague or uninformative prior, ensures that in the absence of prior knowledge or information, all conceivable values of the unknown population parameter are treated with equal probability or weight. In other words, the prior distribution does not favor any specific value or range of values for the parameter, effectively representing a state of maximum uncertainty. This approach is particularly useful when a researcher or analyst wants to maintain objectivity and avoid introducing any bias into the Bayesian analysis, allowing the data to exert the most significant influence on the final inference about the parameter of interest. It is a way to approach Bayesian analysis when one wants to be as neutral as possible regarding the parameter's possible values.The probability function of the uniform prior can be represented by $p\left( \theta \right)$, and its definition is as follows:

2 $$p\left( \theta \right) \propto \sqrt {\frac{n}{{\delta^{2} }}} = c\sqrt {\frac{n}{{\delta^{2} }}}$$

where c represents the constant of proportionality and *n* is the sample size.

The uniform prior is not known for exhibiting the invariance property in Bayesian analysis. However, Jeffrey^24^ introduced an alternative prior distribution, called Jeffrey's prior. This prior is proportional to the Fisher information matrix, a fundamental concept in statistics that quantifies the amount of information contained in a sample from a probability distribution. Jeffrey's prior is particularly valuable because it provides a prior distribution that remains invariant under certain transformations of parameters, making it a useful choice in cases where maintaining invariance is important for robust Bayesian inference. This property ensures that the prior distribution does not change when expressing information in a different parameterization, enhancing the flexibility and applicability of Bayesian analysis. The Jeffrey’s prior for the parameter $\theta$ is given by $p\left( \theta \right) \propto \sqrt {I\left( \theta \right)}$, where $I\left( \theta \right) = - E\left( {\frac{{\partial^{2} }}{{\partial \theta^{2} }}\log f\left( {X/\theta } \right)} \right)$.

The P distribution is denoted by

3 $$p\left( {\theta |x} \right) = \frac{{p\left( {x|\theta } \right)p\left( \theta \right)}}{{\int {p\left( {x|\theta } \right)p\left( \theta \right)d\theta } }}.$$

The The PP distribution in Bayesian analysis combines updated beliefs from the P distribution with the prior distribution to predict future data *Y* probabilistically, considering Bayesian process uncertainty. This aids informed, probabilistic forecasting across various fields. The PP distribution is mathematizied as

4 $$p\left( {y|x} \right) = \int {p\left( {y|\theta } \right)p\left( {\theta |x} \right)d\theta } .$$

In Bayesian analysis, the risk associated with the estimator is mitigated through the use of a LF. In this study, we employed both symmetric and asymmetric LFs to address this aspect.

### Square error loss function

In Bayesian analysis, the symmetric type LF SELF suggested by Gauss^25^. It is commonly employed to measure the disparity between a point estimate (e.g., posterior mean or median) and the true but typically unknown parameter value. It is favored in Bayesian analysis when the objective is to minimize the expected value of the squared difference between the estimate and the true parameter value, exhibiting sensitivity to larger errors. The choice of the LF in Bayesian analysis, including the SELF, depends on the specific problem and data characteristics. The SELF is mathematically defined as

5 $$L\left( {\theta ,\hat{\theta }} \right) = \left( {\theta - \hat{\theta }} \right)^{2} .$$

The Bayesian estimator under SELF

6 $$\hat{\theta }_{{\left( {SELF} \right)}} = E_{\theta /x} \left( \theta \right).$$

The construction of the Bayes estimator by using SELF is included in Appendix A.

### Linex loss function

Varian^26^ was the first to introduce the asymmetric type LF Linex LF (LLF), defined as:

7 $$L\left( {\theta ,\hat{\theta }} \right) = \left( {e^{{c\left( {\theta - \hat{\theta }} \right)}} - c\left( {\theta - \hat{\theta }} \right) - 1} \right).$$

When the estimator $\hat{\theta }$ is employed to estimate the unknown population parameter, it is mathematically represented as:

8 $$\hat{\theta }_{{\left( {LLF} \right)}} = - \frac{1}{c}InE_{\theta /x} \left( {e^{ - c\theta } } \right).$$

## Ranked set sampling

In the RSS procedure, we select *m*^2^ units randomly from the underlying population. The *m*^2^ units are distributed into *m* sets, with *m* similar set size randomly proposed by McIntyre^27^. The study variable *Z* is considered without taking into account the actual measurement and the ordering (ranking) of the m units in each set visually. The The process unfolds systematically by arranging all m sets and sequentially selecting the smallest unit from the first set for measurement. This sequential selection continues as the second smallest unit is chosen from the second set, and so on, until it culminates with the selection of the largest unit from the mth set. This constitutes a single cycle of RSS with a size of m. This cycle is then replicated r times iteratively until the desired sample size, denoted as n, is achieved. The procedure for RSS can be expressed as *Zi*(*j*), *r*, where *i* and *j* signify the sample set and order statistic, respectively, with values ranging from 1 to *m*, while *r* denotes the cycle number. In the case where *c* = 1, the mean and variance of the ranked set sample estimator can be described as follows:

The RSS mean estimator is given as

9 $$\overline{Z}_{{\left( {RSS} \right)}} = \frac{1}{m}\sum\limits_{i = 1}^{m} {Z_{i\left( i \right)} }$$

and the variance of the estimator is given by

10 $${\text{var}} \left( {\overline{Z}_{{\left( {RSS} \right)}} } \right) = \frac{{\delta^{2} }}{m} - \frac{1}{{m^{2} }}\sum\limits_{i = 1}^{m} {\left( {\mu_{\left( i \right)} - \mu } \right)} ,$$

where $\mu$ is the overall mean.

## Median ranked set sampling

Muttlak^28^ introduced the median ranked set sampling (MRSS) method as an estimation technique for the population mean. MRSS exhibits improved performance by minimizing ranking errors compared to the mean estimator used in RSS. Like RSS, MRSS divides the sampling units into *m* sets and arranges them within each set based on the study variable. If *m* is odd, then $\left( {m + 1/2} \right){\text{th}}$ units are picked as a sample from all sets. When *m* is an even, the selection process entails choosing the smallest order units from the two middle sampling units of the first $\left( {m/2} \right){\text{th}}$ set and selecting the highest order units from the two middle sampling units of the remaining $\left( {m/2} \right){\text{th}}$ sets. This method involves completing a single cycle of MRSS with a size of m. If needed, the cycle can be repeated r times to reach the desired sample size $n = mr$.

For a single cycle, the mean estimator for MRSS in case of odd sample size is given by

11 $$\overline{Z}_{{\left( {MRSS} \right)O}} = \frac{1}{m}\left( {\sum\limits_{i = 1}^{m} {Z_{{i\left( {\frac{m + 1}{2}} \right)}} } } \right)$$

and the variance of the estimator is given by

12 $${\text{var}} \left( {\overline{Z}_{{\left( {MRSS} \right)O}} } \right) = \frac{1}{m}\left( {\delta_{{\left( {\frac{m + 1}{2}} \right)}}^{2} } \right).$$

In the case of an even sample size, the population mean estimator of MRSS with one cycle is

13 $$\overline{Z}_{{\left( {MRSS} \right)O}} = \frac{1}{m}\left( {\sum\limits_{i = 1}^{m/2} {Z_{{i\left( \frac{m}{2} \right)}} } + \sum\limits_{i = 1}^{m/2} {Z_{{\frac{m}{2} + i\left( {\frac{m + 1}{2}} \right)}} } } \right)$$

and the variance of the estimator is given by

14 $${\text{var}} \left( {\overline{Z}_{{\left( {MRSS} \right)O}} } \right) = \frac{1}{m}\left( {\delta_{{\left( \frac{m}{2} \right)}}^{2} + \delta_{{\left( {\frac{m + 2}{2}} \right)}}^{2} } \right).$$

## Extreme ranked set sampling

ERSS scheme is the modified form of the RSS suggested by Samawi et al.^29^ and is useful in situations where the collection of *i*th ordered units is more difficult than extreme units. In the ERSS method, a random selection of $m^{2}$ units is made from the underlying population, and they are then randomly distributed into *m* sets of equal size. The units within each set are arranged based on the study variable. When *m* is an odd, the procedure involves selecting the smallest units from the first $\left( {\frac{m - 1}{2}} \right){\text{th}}$ ordered set and the highest units from the following $\left( {\frac{m - 1}{2}} \right){\text{th}}$ ordered sets, ultimately concluding by choosing the median unit from the last set. When *m* is even, the process entails selecting the smallest units from the first $\left( \frac{m}{2} \right){\text{th}}$ ordered units and the highest units from the remaining $\left( \frac{m}{2} \right){\text{th}}$ ordered sets. This constitutes a single iteration, and if necessary, the process is repeated r times to achieve the desired sample size, denoted as *n* = *mr*.

Using ERSS for a single cycle, the mean estimator for an odd sample size is mathematically expressed as

15 $$\overline{Z}_{{\left( {ERSS} \right)O}} = \frac{1}{m}\left( {\sum\limits_{i = 1}^{{\left( {\frac{m - 1}{2}} \right)}} {Z_{i\left( 1 \right)} } + \sum\limits_{i = 1}^{{\left( {\frac{m - 1}{2}} \right)}} {Z_{{\left( {\frac{m - 1}{2}} \right) + i\left( l \right)}} } + Z_{{m\left( {\frac{m + 1}{2}} \right)}} } \right)$$

and the variance of the estimator is given by

16 $${\text{var}} \left( {\overline{Z}_{{\left( {ERSS} \right)O}} } \right) = \frac{1}{{2m^{2} }}\left( {\delta_{\left( 1 \right)}^{2} + \delta_{\left( m \right)}^{2} } \right) + \frac{1}{{l^{2} }}\left( {\delta_{{\left( {\frac{m + 1}{2}} \right)}}^{2} } \right).$$

The mean estimator for ERSS, in the scenario of an odd sample size and a single cycle, is defined as follows:

17 $$\overline{Z}_{{\left( {ERSS} \right)e}} = \frac{1}{m}\left( {\sum\limits_{i = 1}^{{\left( \frac{m}{2} \right)}} {Z_{i\left( 1 \right)} } + \sum\limits_{i = 1}^{{\left( \frac{m}{2} \right)}} {Z_{{\frac{m}{2} + i\left( l \right)}} } } \right)$$

and the variance of the estimator is given by

18 $${\text{var}} \left( {\overline{Z}_{{\left( {ERSS} \right)e}} } \right) = \frac{1}{2m}\left( {\delta_{\left( 1 \right)}^{2} + \delta_{\left( m \right)}^{2} } \right).$$

## EWMA control-chart

Consider a quality characteristic *X* that follows a normal distribution with an unknown location parameter θ and a known scale parameter σ^2^. In this scenario, we can express the probability density function and likelihood function of the random variable *X* as follows:

19 $$f\left( {x;\theta ,\delta^{2} } \right) = \frac{1}{{\sqrt {2\pi } \delta }}\exp \left( { - \frac{1}{{2\delta^{2} }}\left( {x_{i} - \theta } \right)^{2} } \right)$$

20 $$L\left( {\theta ,\delta^{2} ;x_{i} } \right) = \prod\limits_{i = 1}^{n} {f\left( {x_{i} ;\theta ,\delta^{2} } \right)} .$$

Let sample observation $x_{1,} x_{2,} x_{3,} ...x_{n}$ be independently and normally distributed with mean $\theta$ and variance $\delta^{2}$. The statistic of EWMA *CC* is given by

21 $$z_{t} = \lambda \overline{X}_{t} + \left( {1 - \lambda } \right)z_{t - 1} ,\quad t = 1,2,...$$

In this context, $\lambda$ it represents a smoothing constant that adheres to the 0 ≤ $\lambda$ ≤ 1 constraint. The values of $\lambda$ assign weights to both current and past sample observations. When α is set to 1, all weights are exclusively attributed to the current sample. The initial value is initialized to the mean value of the process so that $z_{0} = \theta_{0}$.

The mean and variance of the EWMA statistic is given as

$$E\left( {Z_{t} } \right) = \theta_{0} ,\quad {\text{and}}\quad V\left( {Z_{t} } \right) = \delta^{2} {\raise0.7ex\hbox{$\lambda $} \!\mathord{\left/ {\vphantom {\lambda {2 - \lambda }}}\right.\kern-0pt} \!\lower0.7ex\hbox{${2 - \lambda }$}}\left[ {1 - \left( {1 - \lambda } \right)^{2t} } \right].$$

The control limits *UCL*, *CL* and *LCL* of EWMA *CC* are as follow:

22 $$UCL = E\left( {Z_{t} } \right) + L\sqrt {V\left( {Z_{t} } \right)}$$

23 $$CL = E\left( {Z_{t} } \right)$$

24 $$LCL = E\left( {Z_{t} } \right) - L\sqrt {V\left( {Z_{t} } \right)}$$

In this context, the abbreviations *UCL*, *CL*, and *LCL* correspond to the upper control limit, center line, and lower control limit, respectively. The control constant *L* is employed to modify the predefined average run length ARL_0_. The asymptotic expression for the control limits of the EWMA statistic is presented as follows:

25 $$UCL = E\left( {Z_{t} } \right) + L\delta \sqrt {{\raise0.7ex\hbox{$\lambda $} \!\mathord{\left/ {\vphantom {\lambda {2 - \lambda }}}\right.\kern-0pt} \!\lower0.7ex\hbox{${2 - \lambda }$}}}$$

26 $$CL = E\left( {Z_{t} } \right)$$

27 $$LCL = E\left( {Z_{t} } \right) - L\delta \sqrt {{\raise0.7ex\hbox{$\lambda $} \!\mathord{\left/ {\vphantom {\lambda {2 - \lambda }}}\right.\kern-0pt} \!\lower0.7ex\hbox{${2 - \lambda }$}}} .$$

### Proposed Bayesian-EWMA applying differnt RSS designs

The plotting statistic for the recomended Bayesian EWMA *CC* under different RSS schemes (RSS, MRSS, and ERSS) by using LFs and informative prior for P and PP distribution is defined as:

28 $$Z_{t} = \lambda \left( {\hat{\theta }_{{t\left( {RSS} \right)LF}} } \right) + \left( {1 - \lambda } \right)Z_{t - 1} \quad {\text{and}}\quad Z_{0} = E\left( {\hat{\theta }_{{\left( {RSS} \right)LF}} } \right).$$

where RSS stands for ranked set sampling and LF is used for the loss function, the above structure is work under prior distribution given in “Utilizing normal prior the posterior distribution”.

### Utilizing normal prior the posterior distribution

The P distribution is the resulting probability distribution achieved by combining the likelihood function with a normal prior distribution and mathematically expressed as

29 $$P\left( {\theta |x} \right) = \frac{1}{{\sqrt {2\pi } \sqrt {\frac{{\delta^{2} \delta_{0}^{2} }}{{\delta^{2} + n\delta_{0}^{2} }}} }}\exp \left[ { - \frac{1}{2}\left( {\frac{{\theta - \sum\limits_{i = 1}^{n} {\frac{{x_{i} \delta_{0}^{2} + \theta_{0} \delta_{0}^{2} }}{{\delta^{2} + n\delta_{0}^{2} }}} }}{{\sqrt {\frac{{\delta^{2} \delta_{0}^{2} }}{{\delta^{2} + n\delta_{0}^{2} }}} }}} \right)^{2} } \right].$$

The $P\left( {\theta |x} \right)$ is normally distributed with mean and variance are $\theta_{n}$ and $\delta_{n}^{2}$ respectively and given as $\theta /X \sim N\left( {\theta_{n} ,\delta_{n}^{2} } \right)$, where $\theta_{n} = \frac{{n\overline{x} \delta_{0}^{2} + \delta^{2} \theta_{0} }}{{\delta^{2} + n\delta_{0}^{2} }}$ and $\delta_{n}^{2} = \frac{{\delta^{2} \delta_{0}^{2} }}{{\delta^{2} + n\delta_{0}^{2} }}$. The construction of the P and PP distribution under normal prior is derived in Suppl. Appendix B.

The control limits for the Bayesian EWMA CC, established using both P and PP distributions and factoring in various LFs within the RSS schemes, are presented below.

#### Under RSS, control limits utilizing SELF

The SELF serves as a LF that facilitates the establishment of control limits for Bayesian-EWMA *CC*s through the utilization of RSS schemes. The estimator $\hat{\theta }$ based on SELF is mathematizied as:

30 $$\hat{\theta }_{SELF} = \frac{{n\overline{x}_{{(RSS_{i} )}} \delta_{0}^{2} + \delta^{2} \theta_{0} }}{{\delta^{2} + n\delta_{0}^{2} }}$$

The properties of the estimator $\hat{\theta }_{SELF}$ are mathematically expressed as:

31 $$E\left( {\hat{\theta }_{SELF} } \right) = \frac{{n\theta_{1} \delta_{0}^{2} + \delta^{2} \theta_{0} }}{{\delta^{2} + n\delta_{0}^{2} }}$$

and

32 $${\text{var}} \left( {\hat{\theta }_{SELF} } \right) = \frac{{n\delta_{{(RSS_{i} )}}^{2} \delta_{0}^{4} }}{{\delta^{2} + n\delta_{0}^{2} }}.$$

In Suppl. Appendix C, we explained the process of deriving the Bayes estimator using SELF under RSS strategies for both the P and PP distributions. The asymptotic control limits of the EWMA CC under the Bayesian approach with RSS and SELF are formulated as follows:

33 $$UCL_{{RSS_{i} }} = E\left( {\hat{\theta }_{SELF} } \right) + L\sqrt {{\text{var}} \left( {\hat{\theta }_{SELF} } \right)} \sqrt {{\raise0.7ex\hbox{$\lambda $} \!\mathord{\left/ {\vphantom {\lambda {2 - \lambda }}}\right.\kern-0pt} \!\lower0.7ex\hbox{${2 - \lambda }$}}}$$

34 $$CL_{{RSS_{i} }} = E\left( {\hat{\theta }_{SELF} } \right)$$

35 $$LCL_{{RSS_{i} }} = E\left( {\hat{\theta }_{SELF} } \right) - L\sqrt {{\text{var}} \left( {\hat{\theta }_{SELF} } \right)} \sqrt {{\raise0.7ex\hbox{$\lambda $} \!\mathord{\left/ {\vphantom {\lambda {2 - \lambda }}}\right.\kern-0pt} \!\lower0.7ex\hbox{${2 - \lambda }$}}} .$$

$$\begin{aligned} {\text{where}} \quad i = 1,2,3.\quad & RSS_{1} = RSS \\ & RSS_{2} = MRSS \\ & RSS_{3} = ERSS \\ \end{aligned}$$.

#### Under LLF, control limits utilizing RSS schemes

The LLF serves as an instance of an asymmetric LFs applied in establishing control limits for the EWMA CC within the Bayesian framework when employing RSS schemes. The estimator of $\hat{\theta }_{LLF}$ is defined as

36 $$\hat{\theta }_{LLF} = \frac{{n\overline{x}_{{(RSS_{i} )}} \delta_{0}^{2} + \delta^{2} \theta_{0} }}{{\delta^{2} + n\delta_{0}^{2} }} - \frac{C\prime }{2}\delta_{n}^{2} .$$

The properties of the estimator $\hat{\theta }_{LLF}$ are given below:

37 $$E\left( {\hat{\theta }_{LLF} } \right) = \frac{{n\theta_{1} \delta_{0}^{2} + \delta^{2} \theta_{0} }}{{\delta^{2} + n\delta_{0}^{2} }} - \frac{C\prime }{2}$$

and

38 $${\text{var}} \left( {\hat{\theta }_{LLF} } \right) = \frac{{n\delta_{{(RSS_{i} )}}^{2} \delta_{0}^{4} }}{{\left( {\delta^{2} + n\delta_{0}^{2} } \right)^{2} }}.$$

The asymptotic control limits of Bayesian-EWMA CC with RSS schemes and LLF loss function are defined as

39 $$UCL_{{RSS_{i} }} = E\left( {\hat{\theta }_{LLF} } \right) + L\sqrt {{\text{var}} \left( {\hat{\theta }_{LLF} } \right)} \sqrt {{\raise0.7ex\hbox{$\lambda $} \!\mathord{\left/ {\vphantom {\lambda {2 - \lambda }}}\right.\kern-0pt} \!\lower0.7ex\hbox{${2 - \lambda }$}}}$$

40 $$CL_{{RSS_{i} }} = E\left( {\hat{\theta }_{LLF} } \right)$$

41 $$LCL_{{RSS_{i} }} = E\left( {\hat{\theta }_{LLF} } \right) - L\sqrt {{\text{var}} \left( {\hat{\theta }_{LLF} } \right)} \sqrt {{\raise0.7ex\hbox{$\lambda $} \!\mathord{\left/ {\vphantom {\lambda {2 - \lambda }}}\right.\kern-0pt} \!\lower0.7ex\hbox{${2 - \lambda }$}}} .$$

### PP distribution using normal distribution

In this section, the EWMA CC utilizing Bayesian theory has been constructed using PP distribution. Let the future observation of size *k* are $y_{1} ,y_{2} ,....,y_{k}$, then the PP distribution $Y|X$is derived as:

42 $$p\left( {y|x} \right) = \frac{1}{{\sqrt {2\pi \delta_{1}^{2} } }}\exp \left\{ { - \frac{1}{{2\delta_{1}^{2} }}\left( {Y - \theta_{n} } \right)^{2} } \right\}.$$

where $\delta_{1}^{2} = \delta^{2} + \frac{{\delta^{2} \delta_{0}^{2} }}{{\delta^{2} + n\delta_{0}^{2} }}$, the Bayesian-EWMA control limits for PP distribution using various loss functions by using RSS schemes are given in subsection “Under LLF, control limits utilizing RSS schemes”.

#### Under LLF, control limits utilizing RSS schemes

The Bayesian-EWMA CC under asymmetric LLF and with different RSS designs, the estimator $\hat{\theta }_{LLF}$ is mathematized as

43 $$\hat{\theta }_{LLF} = \frac{{n\overline{x}_{{(RSS_{i} )}} \delta_{0}^{2} + \delta^{2} \theta_{0} }}{{\delta^{2} + n\delta_{0}^{2} }} - \frac{C\prime }{2}\tilde{\delta }_{1}^{2}$$

where $$\tilde{\delta }_{1}^{2} = \frac{{\delta^{2} }}{k} + \frac{{\delta^{2} \delta_{0}^{2} }}{{\delta^{2} + n\delta_{0}^{2} }}$$.

The properties of $\hat{\theta }_{LLF}$ described as:

44 $$E\left( {\hat{\theta }_{LLF} } \right) = \frac{{n\theta_{1} \delta_{0}^{2} + \delta^{2} \theta_{0} }}{{\delta^{2} + n\delta_{0}^{2} }} - \frac{C\prime }{2}\tilde{\delta }_{1}^{2}$$

and

45 $${\text{var}} \left( {\hat{\theta }_{SELF} } \right) = \frac{{n\delta_{{(RSS_{i} )}}^{2} \delta_{0}^{4} }}{{\left( {\delta^{2} + n\delta_{0}^{2} } \right)^{2} }}.$$

The control limits for Bayesian-EWMA CC under LLF and utilizing various RSS schemes are given below.

46 $$UCL_{{RSS_{i} }} = E\left( {\hat{\theta }_{LLF} } \right) + L\sqrt {{\text{var}} \left( {\hat{\theta }_{LLF} } \right)} \sqrt {{\raise0.7ex\hbox{$\lambda $} \!\mathord{\left/ {\vphantom {\lambda {2 - \lambda }}}\right.\kern-0pt} \!\lower0.7ex\hbox{${2 - \lambda }$}}}$$

47 $$CL_{{RSS_{i} }} = E\left( {\hat{\theta }_{LLF} } \right)$$

48 $$LCL_{{RSS_{i} }} = E\left( {\hat{\theta }_{LLF} } \right) - L\sqrt {{\text{var}} \left( {\hat{\theta }_{LLF} } \right)} \sqrt {{\raise0.7ex\hbox{$\lambda $} \!\mathord{\left/ {\vphantom {\lambda {2 - \lambda }}}\right.\kern-0pt} \!\lower0.7ex\hbox{${2 - \lambda }$}}}$$

## Simulation study

The The assessment of the Bayesian EWMA CCs performance in this study adheres to standard quality control methodologies, relying on ARL and SDRL as primary performance indicators. These metrics gauge the control charts efficiency across diverse ranked-based sampling designs while considering two distinct LFs and the inclusion of informative priors. Specifically, ARL_0_ and SDRL_0_ quantify the ARL and SDRL under normal, in-control conditions, while ARL_1_ and SDRL_1_ reflect scenarios where the process is out of control. The effectiveness of a CC is typically judged by its capacity to achieve a smaller ARL_1_ for a specific shift. To compute efficiency metrics, the Monte Carlo simulation method is employed, encompassing 20,000 iterations. The control constant is adjusted to achieve a predetermined ARL_0_ under varying LFs and different values of $\lambda$, such as $\lambda$ = 0.10 and 0.25. The simulation study encompasses the use of both the P and PP distributions to anlyze Bayesian EWMA CC using ranked-based sampling designs with sample size *n* = 5 and assumed standard normal distribution as prior distribution. Detailed steps for simulating ARL and SDRL are provided below.

### Step 1: for in-control process

i. We choice a specific value of $\lambda$ and *L* smoothing constants for fixed ARL_0_.
ii. Draw a sample with a RSS stratigies for an in-control process from normal distribution such that $X \sim N\left( {E\left( {\hat{\theta }} \right),\delta } \right)$.
iii. Computing the mean and standard deviation for the P distribution under different LFs, while assuming standard normal distributions for the sampling and prior distributions.
iv. Based on Bayesian theory, the proposed Bayesian EWMA statistics are computed to evaluate the process according to the projected strategy.
v. Considered Implemented the CC methodology, and in case of out-of-control process signals, documented the number of subgroups as the in-control run length.length.
vi. Repeating the steps 100,000 times to estimate in-control ARL_0_.

### Step 2: evaluation of the out-of-control process

i. Draw a ranked set sample of size *n* from the shifted process by utilizing normal distribution, such that $X \sim N\left( {E\left( {\hat{\theta }} \right) + \partial \frac{\delta }{\sqrt n },\delta } \right)$, where $\partial$ represents the shift in the process mean.
ii. Compute the recommended Bayesian-EWMA statistic and assess the process based on the proposed study design.
iii. If out-of-control signals are detected during the process, implement the CC method and record the number of run lengths for the out-of-control process.
iv. Repeating the steps 100,000 times to estimate out-of-control ARL.

## Results, discussion and findings

**Figure 1:**
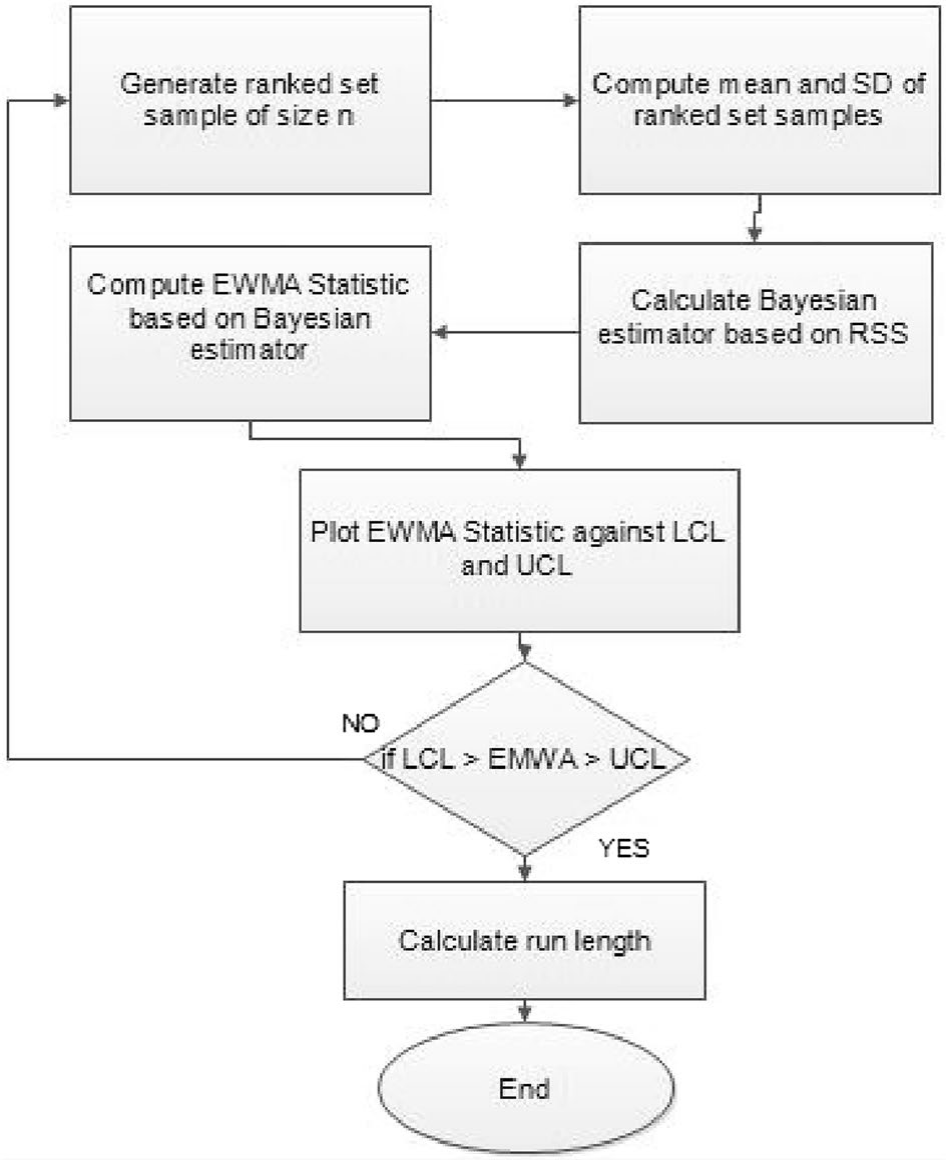
Flow chart for the suggested CC applying RSS schems.

**Figure 2:**
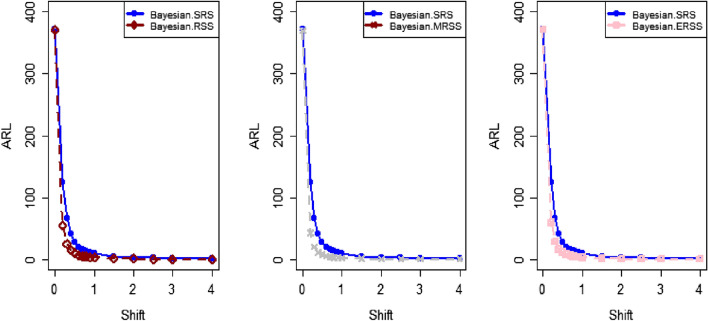
Employing SELF, ARL plots for the recommended CC applying the RSS, MRSS, and ERSS schemes.

**Figure 3:**
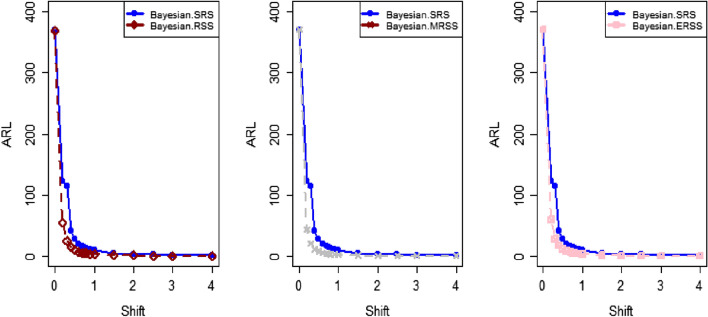
Utlizing LLF, ARL plots of the suggested *CC* under RSS, MRSS, and ERSS for P distribution.

The study results, as presented in Tables 1, 2, 3, 4, 5 and 6, provide a comprehensive comparison of the Bayesian EWMA CC utilizing informative prior distributions and two distinct LFs across various ranked-based sampling designs. These findings emphasize the charts performance in comparison to the traditional Bayesian-EWMA CC designed for SRS. The analysis illuminates the strengths of the proposed Bayesian EWMA CC under diverse conditions, underscoring the importance of ranked-based sampling methods and the choice of LFs. In summary, this study demonstrates the considerable potential of the proposed method to significantly enhance process monitoring and improve quality control practices. The findings highlight a significant advantage of the suggested Bayesian EWMA CC for RSS, MRSS, and ERSS exhibits superior performance compared to the Bayesian EWMA CC for SRS at every shift. For example, Table 1 gives the values of ARLs and SDRLs of proposed Bayesian EWMA for RSS schemes (i.e. RSS, MRSS, ERSS) and Bayesian-EWMA *CC* by using SRS for P and PP distribution utilizing SELF as 371, 55.66, 10.36, 4.69, 1.29, 1 for RSS, 369.42, 43.99, 8.53, 3.95, 1.16, 1 for MRSS, 371, 60.05, 11.80, 5.29, 1.39, 1 for ERSS and Bayesian EWMA CC for SRS values for the same shift are 371.62, 125.58, 28.35, 13.41, 4.16, 2.12 at $\lambda$ = 0.10. The results suggest that, for every shift value, the proposed method consistently exhibits significantly lower ARL values compared to the aviable Bayesian CC. This observation underscores the superior performance of the proposed Bayesian EWMA CC when applied to RSS strategies in comparison to the existing Bayesian EWMA CC designed for SRS. For LLF and at $\lambda$ = 0.25, we considered Table 4 which show that the ARL results for the suggested method are 371.79, 85.76, 13.91, 5.59, 1 for RSS, 370.89, 66.78, 11.25, 4.59, 1 for MRSS, and 369.55, 100.52, 15.71, 6.33, 1 for ERSS, the values of ARL for Bayesian-EWMA SRS are 371.05, 179.81, 28.05, 15.89, 1.66. The run length profiles, which represent the number of consecutive samples required to detect an out-of-control condition, consistently exhibit smaller values for the suggested CC when compared to the considered CC. This trend suggests that the proposed CC outperforms the considered CC in terms of its ability to detect process deviations more quickly and efficiently. To further illustrate this efficiency, Figs. 1, 2 and 3 in the ARL demonstrate the effectiveness of the proposed CC when utilizing various RSS schemes. These figures provide visual evidence of the proposed CC's effectiveness in different scenarios. The comparison of the variances obtained through simulations under the considered sampling schemes is presented in Table D1 of Suppl. Appendix D. The results show that the variances under the RSS schemes are consistently lower than those of SRS. This comparison reinforces the efficiency of the suggested CC based on RSS schemes. In the following section, we will delve into the key findings and implications of the proposed CC.

- The The appraisal of the offered CC involved analyzing its performance across various smoothing constant (λ) values. Tables 1 and 2 provided comprehensive results on the ARL and SDRL for the both P and PP distributions, with a normal prior and the SELF. These tables illuminated how the Bayesian-EWMA CCs efficiency varied with different λ values. The key finding was that the Bayesian EWMA CC achieved its optimal performance at the smallest λ value. In essence, when the smoothing constant was set to its minimum, the CC exhibited the highest sensitivity in detecting process deviations. This observation underscores the critical role of selecting an appropriate λ value, as a smaller λ enhances the ability to detect process changes promptly—an essential aspect of quality control and process monitoring.For example, at $ARL_{0} = 370$, δ = 0.30, and $\lambda$ = 0.1, the ARL value is 25.63 and for $\lambda$ = 0.25, the ARL result is 40.81 for RSS, 21.10 and 31.19 are the ARL values using MRSS and the ARL results using ERSS is 29.31 and 46.44.
- With an increase in the magnitude of the shift, the ARL values of the suggested Bayesian EWMA CC for RSS schemes decrease faster than the considered Bayesian-EWMA *CC*. For example, from Tables 1 and 2, we can observe that at $ARL_{0} = 370$, and with $\lambda$ = 0.1, the ARL value for δ = 0.20 is 55.66 and for δ = 0.50 is 10.36 using RSS, 43.99 and 8.53 utilizing MRSS and under the similar condition, the ARL result using ERSS is 60.05 and 11.80, which shows that the suggested design is unbiased.
- The outcomes presented in Tables 3 and 4 show the run length results under LLF for Pdistribution for $ARL_{0} = 370$, $\lambda = 0.10$ and for δ = 0.50 the ARL value is 10.43 and 13.91 for $\lambda = 0.25$, 8.47 and 11.25 for MRSS and in the same case, the run length outcomes using ERSS is 11.81 and 15.71, which clearly shows that as the value of $\lambda$ increases the performance of the recommended CC distribution decreases.
- The results for PP distribution under LLF are presented in Tables 5 and 6, which show that the ARL values at $ARL_{0} = 370$, δ = 0.20 and 54.68 and the ARL values for $\lambda = 0.25$ is 85.76 using RSS, In the identical situation the ARL results are 44.30 and 71.03. Under ERSS the ARL outcomes are 62.82 and 97.99. The results presented in Tables 3, 4, 5 and 6 indicate that the proposed Bayesian-EWMA *CC* for the Pdistribution utilizing LLF yields a similar performance to the PP distribution under LLF.
- After a comprehensive analysis of the outcomes provided in Tables 1, 2, 3, 4, 5 and 6, which detail the performance of the proposed CC utilizing both P and PP distributions, and considering both SELF and LLF, it becomes apparent that the Bayesian EWMA CC incorporating MRSS excels in terms of efficiency when it comes to detecting shifts in the monitored process. When compared to other sampling design strategies, the offered CC utilizing MRSS consistently demonstrates a higher level of effectiveness in promptly and accurately identifying and responding to shifts in the process. This superior performance suggests that MRSS is a particularly suitable approach when aiming to enhance the efficiency of process monitoring and quality control through the Bayesian EWMA CC framework.

**Table 1:** Under SELF, AEL and SDRL outcomes of Bayesian CC with $\lambda$ = 0.10.

Shift	Bayes-EWMA SRS		Bayes-EWMA RSS		Bayes-EWMA MRSS		Bayes-EWMA ERSS	
	ARL	SDRL	ARL	SDRL	ARL	SDRL	ARL	SDRL
	*L* = 2.7042		*L* = 2.9142		*L* = 2.7132		*L* = 2.7172	
0.00	371.82	367.80	371.05	368.24	369.42	366.98	371.33	369.32
0.20	125.58	114.98	55.66	50.69	43.99	38.78	60.05	55.88
0.40	41.68	32.78	15.23	11.23	12.51	8.89	17.52	13.05
0.60	20.98	13.49	7.65	4.92	6.25	3.92	8.58	5.64
0.75	14.72	8.30	5.27	3.23	4.35	2.55	5.90	3.68
0.80	13.41	7.14	4.69	2.84	3.95	2.28	5.29	3.20
1.00	9.78	4.50	3.37	1.89	2.80	1.52	3.69	2.11
2.00	4.16	1.20	1.29	0.50	1.16	0.37	1.39	0.58
2.50	3.31	0.84	1.07	0.26	1.02	0.15	1.12	0.33
4.00	2.12	0.38	1	0	1	0	1	0

**Table 2:** ARL and SDRL outcomes of Bayesian CC with $\lambda$ = 0.25 based on SELF.

Shift	Bayes-EWMA SRS		Bayes-EWMA RSS		Bayes-EWMA MRSS		Bayes-EWMA ERSS	
	ARL	SDRL	ARL	SDRL	ARL	SDRL	ARL	SDRL
	*L* = 2.8987		*L* = 2.9042		*L* = 2.9052		*L* = 2.9129	
0.00	369.49	364.82	371.34	367.87	370.18	368.76	371.01	368.98
0.20	178.20	175.14	86.62	84.06	70.45	66.92	98.59	96.41
0.40	63.11	58.20	21.77	18.64	17.19	13.96	26.17	22.86
0.60	28.45	24.57	9.42	6.87	7.58	5.16	11.07	8.24
0.75	17.97	13.87	6.20	3.99	5.07	3.13	7.14	4.83
0.80	15.71	11.75	5.53	3.47	4.53	2.71	6.31	4.08
1.00	10.22	6.77	3.78	2.17	3.11	1.71	4.26	2.49
2.00	3.46	1.33	1.37	0.56	1.21	0.43	1.49	0.63
2.50	2.66	0.86	1.10	0.30	1.04	0.19	1.16	0.38
4.00	1.66	0.50	1	0	1	0	1	0

**Table 3:** Using LLF, run length profiles of Bayesian CC for P distribution with $\lambda$ = 0.10.

Shift	Bayes-EWMA SRS		Bayes-EWMA RSS		Bayes-EWMA MRSS		Bayes-EWMA ERSS	
	ARL	SDRL	ARL	SDRL	ARL	SDRL	ARL	SDRL
	*L* = 2.7047		*L* = 2.7331		*L* = 2.7245		*L* = 2.7142	
0.00	370.63	368.13	369.39	368.11	371.09	367.67	371.11	368.77
0.20	123.94	115.00	54.97	49.58	44.18	38.65	60.39	54.44
0.40	41.33	32.49	15.51	11.25	12.49	8.72	17.55	13.35
0.60	20.95	13.50	7.65	4.99	6.31	3.93	8.58	5.74
0.75	14.79	8.35	5.35	3.24	4.38	2.57	5.88	3.62
0.80	13.38	7.17	4.80	2.81	3.96	2.27	5.30	3.27
1.00	9.79	4.49	3.33	1.85	2.82	1.52	3.71	2.13
2.00	4.18	1.20	1.30	0.51	1.16	0.38	1.39	0.57
2.50	3.31	0.84	1.07	0.26	1.02	0.15	1.11	0.33
4.00	2.13	0.383	1	0	1	0	1	0

**Table 4:** Using LLF, run length outcomes of Bayesian CC for P distribution with $\lambda$ = 0.25.

Shift	Bayes-EWMA SRS		Bayes-EWMA RSS		Bayes-EWMA MRSS		Bayes-EWMA ERSS	
	ARL	SDRL	ARL	SDRL	ARL	SDRL	ARL	SDRL
	*L* = 2.9050		*L* = 2.9142		*L* = 2.9183		*L* = 2.9101	
0.00	371.05	368.88	371.79	364.28	370.77	368.76	369.55	364.62
0.20	179.81	175.30	87.29	86.83	66.78	64.33	100.52	96.45
0.40	64.00	59.50	22.07	18.89	17.53	14.40	25.58	22.83
0.60	28.54	24.33	9.63	6.96	7.78	5.44	10.99	8.33
0.75	18.11	14.02	6.28	4.09	5.08	3.12	7.08	4.80
0.80	15.89	11.94	5.59	3.55	4.59	2.74	6.33	4.06
1.00	10.27	6.77	3.76	2.11	3.12	1.68	4.23	2.48
2.00	3.46	1.33	1.37	0.55	1.22	0.43	1.48	0.63
2.50	2.64	0.85	1.10	0.31	1.03	0.19	1.16	0.38
4.00	1.66	0.50	1	0	1	0	1	0

**Table 5:** Using LLF, the run length values of Bayesian CC for PP distribution with $\lambda$ = 0.10.

Shift	Bayes-EWMA SRS		Bayes-EWMA RSS		Bayes-EWMA MRSS		Bayes-EWMA ERSS	
	ARL	SDRL	ARL	SDRL	ARL	SDRL	ARL	SDRL
	*L* = 2.7018		*L* = 2.7191		*L* = 2.7238		*L* = 2.7351	
0.00	371.50	368.69	370.39	367.18	369.89	366.76	371.39	369.01
0.20	122.45	113.13	54.68	48.80	44.30	39.35	62.82	57.15
0.40	41.41	32.72	15.53	11.34	12.68	8.98	17.88	13.21
0.60	21.08	13.64	7.71	4.96	6.25	3.89	8.83	5.83
0.75	14.73	8.23	5.30	3.22	4.36	2.56	6.04	3.73
0.80	13.33	7.17	4.75	2.85	3.94	2.29	5.46	3.29
1.00	9.65	4.44	3.37	1.87	2.80	1.50	3.81	2.17
2.00	4.18	1.20	1.29	0.50	1.15	0.37	1.40	0.58
2.50	3.30	0.84	1.07	0.26	1.02	0.16	1.12	0.33
4.00	2.13	0.38	1	0	1	0	1	0

**Table 6:** Using LLF, the run length outcomes of Bayesian-EWMA *CC* for PP distribution with $\lambda$ = 0.25.

Shift	Bayes-EWMA SRS		Bayes-EWMA RSS		Bayes-EWMA MRSS		Bayes-EWMA ERSS	
	ARL	SDRL	ARL	SDRL	ARL	SDRL	ARL	SDRL
	L = 2.8986		L = 2.8942		L = 2.8982		L = 2.9182	
0.00	370.23	368.87	371.86	368.37	370.89	368.30	368.97	367.65
0.20	177.79	174.80	85.76	82.63	71.03	64.97	97.99	94.26
0.40	63.21	58.43	22.07	18.80	17.17	14.01	25.63	22.35
0.60	28.35	24.16	9.41	6.85	7.60	5.24	11.32	8.37
0.75	18.04	14.01	6.23	4.02	5.03	3.10	7.13	4.80
0.80	15.79	11.87	5.50	3.45	4.46	2.67	6.33	4.06
1.00	10.23	6.71	3.75	2.14	3.10	1.66	4.26	2.54
2.00	3.46	1.33	1.36	0.55	1.20	0.42	1.50	0.64
2.50	2.65	0.86	1.09	0.30	1.03	0.19	1.16	0.38
4.00	1.66	0.50	1	0	1	0	1	0

## Real data application

In the field of SPC, it is a customary approach for researchers to evaluate the effectiveness of CC by employing a combination of simulated and real datasets. For this study, we utilized a dataset obtained from Montgomery^30^, consisting of flow width during the hard-bake process. The primary focus of this study is to assess the performance of the newly proposed Bayesian CCs within the context of ranked-based sampling strategies. Our primary objective revolves around the development of a robust statistical CC tailored to effectively monitor variations in the flow width of the resist. The flow width measurements were taken in microns at hourly intervals. The dataset comprises 45 samples, each consisting of measurements from five wafers, totaling 225 observations. It's important to note that we designate the first 30 samples as representing the in-control process, constituting the phase-I dataset, which includes 150 observations. Control limits are established based on this phase-I dataset, assuming a standard normal distribution. The remaining 15 samples are categorized as part of the out-of-control process, involving an ascending shift of 0.017 added to the central process mean, thus forming the phase-II dataset. The subsequent steps delineate the procedure for implementing the proposed Bayesian EWMA CC under various ranked-based sampling schemes.

Step 1: Select twenty-five observations by SRS from 150 control observations and divide them into five groups of equal size randomly at the time *t*. Note that the sample from the in-control dataset is generated for *t* = 1, 2, …., 30.

Step 2: Rank the observations within each group and select the five observations for measurement purposes by following the sampling schemes such as RSS, MRSS, and ERSS. For instance, the diagonal elements will be selected for the RSS scheme.

Step 3: Compute the sample means such as $\overline{Z}_{SRS}$, $\overline{Z}_{RSS}$, $\overline{Z}_{MRSS}^{O}$ and $\overline{Z}_{ERSS}^{O}$ for $t = 1,2,...,45$. The shifted dataset is used for *t* = 31, 32, ..., 45.

Step 4: Compute the Bayes estimator and its standard deviation using different loss functions such as (SELF and LLF) from the sample means ($\overline{Z}_{SRS}$, $\overline{Z}_{RSS}$, $\overline{Z}_{MRSS}^{O}$ and $\overline{Z}_{ERSS}^{O}$).

Step 5: Calculate the Bayesian-EWMA statistic by following the design of the *CC* and plotting the graph with the control limits.

**Figure 4:**
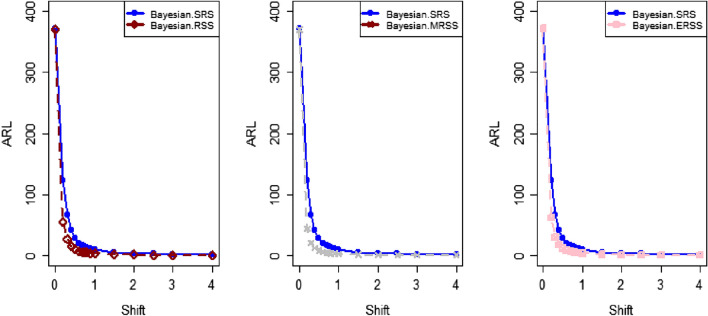
Using LLF, ARL plots of the suggested CC under different RSS strategies for PP distribution.

**Figure 5:**
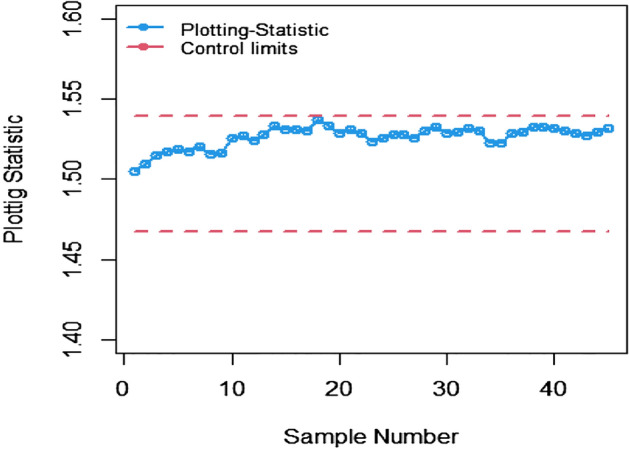
Applying SRS, Bayesian EWMA CC with SELF.

**Figure 6:**
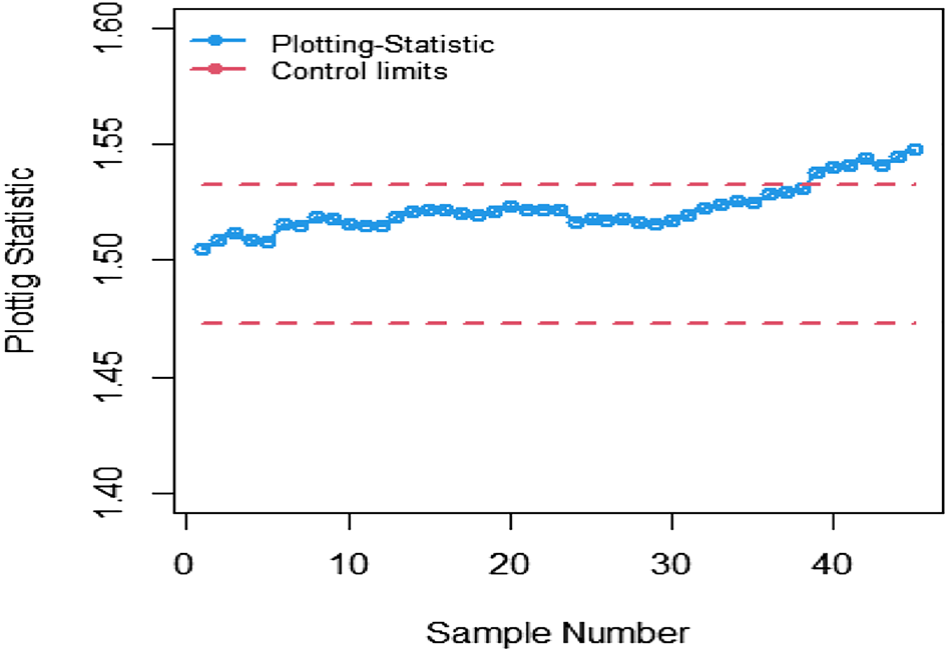
Under RSS, Bayes EWMA CC utilizing SELF.

**Figure 7:**
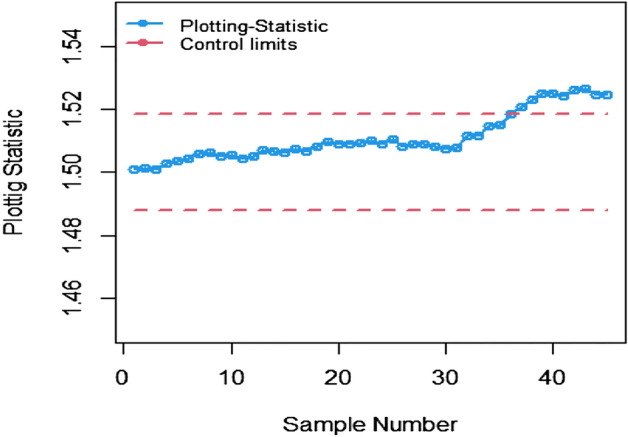
Under MRSS, Bayes EWMA CC applying SELF.

**Figure 8:**
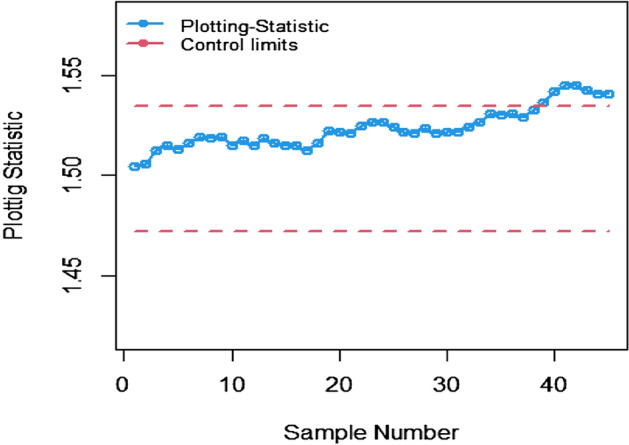
Utilizin SELF, Bayesian EWMA CC using ERSS.

The Bayes estimator using SELF for P and PP distribution utilizing informative prior is 1.5056 for the in-control dataset. The control limits such as LCL and UCL are 1.46 and 1.54 respectively using SRS. The LCL and UCL for RSS are 1.47 and 1.53, LCL and UCL for MRSS are 1.48 and 1.51 respectively. The respective control limits for the Bayesian-EWMA for ERSS are 1.47 and 1.53. Figure 5 presents the Bayesian-EWMA*CC* under SRS for P and PP distribution in which all the points are within the control limits. Figures 6, 7, and 8 present visual representations of the Bayesian EWMA CC as applied to various RSS strategies, including RSS, MRSS, and ERSS schemes. These charts serve as visual indicators of instances where the process exhibits out-of-control behavior, with such deviations occurring at the 37th, 34th, and 36th samples, respectively. Upon a comprehensive analysis of all Figs. 1, 2, 3, 4, 5, 6, 7 and 8, it becomes evident that the Bayesian EWMA CC, particularly when employed within the RSS frameworks displays superior efficiency in promptly identifying out-of-control signals in comparison to the pre-existing Bayesian CC. These findings underscore the enhanced capabilities of the proposed CC in the context of process monitoring and quality control practices.

## Conclusion

The present study uses the ranked-based sampling designs to improve Bayesian CC. The suggested CC applying P and PP distribution is constructed to evaluate the variations in the production process. The run length findings were adapted to asses the effectiveness of recomended CC using RSS schemes. The simulation study was conducted to assess the comparative effectiveness of the Bayesian CC using RSS schemes in contrast to the established Bayesian EWMA CC using SRS. The results presented in Tables 1, 2, 3, 4, 5 and 6 consistently indicate that the proposed Bayesian CC, when employed in conjunction with RSS, MRSS, and ERSS and utilizing both LFs for both P and PP distributions, outperforms the traditional Bayesian EWMA CC based on SRS in terms of its ability to promptly and accurately detect out-of-control signals. This underscores the excellence of the suggested Bayesian CC in the realm of quality control and process monitoring. Furthermore, the superiority of the proposed CC becomes evident when examining the ARL plots (Fig. 1, 2, and 3). These plots provide a visual representation of how quickly the CC can detect out-of-control signals. Additionally, apart from the ARL plots, we demonstrate the effectiveness of the recommended CC through a practical example involving the hard bake process in semiconductor manufacturing. The results of this real-world application affirm that the Bayesian EWMA CC, when deployed under RSS schemes, displays a heightened sensitivity in promptly identifying out-of-control signals in comparison to its counterpart operating under the SRS framework. This heightened sensitivity underscores the practical advantages of implementing the proposed Bayesian EWMA CC in the context of quality control and process monitoring within semiconductor manufacturing.

## Limitations of the study

In scenarios involving large sample sizes, the implementation of Bayesian CCs under RSS designs may encounter computational challenges. This complexity stems from the necessity of conducting Bayesian updates for both the process mean and variance at each individual sample point. As a result, this procedure can become computationally intensive and time-consuming due to the substantial computational workload it entails, rendering it resource-intensive.

Another limitation is that the Bayesian approach specifies prior distributions for the process mean and variance. If the prior distributions are not chosen carefully, the performance of the *CC* may be affected. Moreover, the selection of prior distributions can be subjective and may require expert knowledge, potentially introducing bias into the analysis.

Finally, using RSS in the Bayesian-EWMA *CC* assumes that the data is exchangeable. If the data is not exchangeable, the *CC* may not perform as expected, and the results may be unreliable. Therefore, it is important to assess the exchangeability of the data before using RSS in the Bayesian-EWMA *CC*.

## Future recommendations

The proposed Bayesian EWMA CCs under RSS schemes can be applied to other memory-type CCs. This approach is also versatile enough to handle distributions beyond the normal distribution, including Poisson or binomial distributions, with the requirement of appropriate adjustments to the likelihood function in Bayesian updating. This extension of the approach to cover diverse types of CCs and non-normal distributions has the potential to enhance the effectiveness and efficiency of quality control processes across various industries, such as healthcare, finance, and manufacturing.

### Supplementary Information

Supplementary Information.

## Data Availability

The corresponding author is available to provide the datasets used or analyzed in this study upon a reasonable request.
