# Supplementary material for: Monitoring of manufacturing process using bayesian EWMA control chart under ranked based sampling designs
Source: Sci Rep. 2023 Oct 25;13:18240. doi: 10.1038/s41598-023-45553-x (PMC10600236; doi:10.1038/s41598-023-45553-x)
Supplement: Supplementary file 1 — Supplementary Information. [file 41598_2023_45553_MOESM1_ESM.docx]

# Appendix A

###### Based on the SELF, the process involves constructing Bayes estimators under this specific loss function.

- 1. **Under SELF Derivation of Bayes estimator**

The mathematical expression for SELF is follow as,

$L\left(\theta,\hat{\theta}\right)=\left(\theta-\hat{\theta}\right)^{2}$,

Applying expectation on both sides, we have

$E(L\left(\theta,\hat{\theta}\right))=E\left(\theta-\hat{\theta}\right)^{2}$,

After taking a partial derivative with respect toand setting it equal to zero, we have

$\frac{\partial E(L\left(\theta,\hat{\theta}\right))}{\partial\hat{\theta}}=2E\left(\theta-\hat{\theta}\right)(-1)=0$,

after simplifying the expression, the resulting Bayes estimator under SELF is

$\hat{\theta}_{SELF}=E_{\theta|\mathbf{x}}(\theta)$. **(A1)**

If *Y* represents the predictor variable and Ŷ represents its estimate, SELF can be defined as follows:

$L\left(y,\hat{\theta}\right)$=$\left(y-\hat{\theta}\right)^{2}$,

Similarly the estimator $\hat{\theta}$ which minimize the $L\left(y,\hat{\theta}\right)$is given as,

$\hat{\theta}_{SELF1}=E_{y|\mathbf{x}}(y)$. **(A2)**

# Appendix-B

- 1. **Under normal prior the Posterior distribution**

The P distribution can be obtained by employing the following equations,

$p(\theta)=\frac{1}{\sigma_{0}\sqrt{2\pi}}exp\left[-\frac{1}{2}\left(\frac{\theta-\theta_{0}}{\sigma_{0}}\right)^{2}\right]$,

$$p(\mathbf{x}|\theta)=L(\mathbf{x}/\theta)=\prod_{i=1}^{n} \frac{1}{\sqrt{2\pi\sigma^{2}}}\exp(-\frac{1}{2\sigma^{2}}(x_{i}-\theta)^{2})$$

and

$p(\mathbf{x}|\theta)\times p(\theta)$=$\frac{1}{\sigma_{0}\sigma^{n}(2\pi)^{\frac{n+1}{2}}}\exp\left\{-\frac{1}{2}\left(\frac{\sum_{i=1}^{n} (x_{i}-\theta)^{2}}{\sigma^{2}}-\frac{(\theta-\theta_{0})^{2}}{\sigma_{0}^{2}}\right)\right\}$

$$p(\mathbf{x}|\theta)\times p(\theta)=\frac{1}{\sigma_{0}\sigma^{n}(2\pi)^{\frac{n+1}{2}}}\exp\left[-\frac{1}{2\sigma^{2}\sigma_{0}^{2}}\left\{\sigma_{0}^{2}\left(\sum_{i=1}^{n} x_{i}^{2}+n\theta^{2}-2\theta\sum_{i=1}^{n} x_{i}\right)+\sigma^{2}\left(\theta^{2}+\theta_{0}^{2}-2\theta\theta_{0}\right)\right\}\right]$$

$p(\mathbf{x}|\theta)\times p(\theta)=\frac{1}{\sigma_{0}\sigma^{n}(2\pi)^{\frac{n+1}{2}}}\exp\left[-\frac{1}{2\frac{\sigma^{2}\sigma_{0}^{2}}{\sigma^{2}+n\sigma_{0}^{2}}}\left\{\left(\theta-\frac{\sum_{i=1}^{n} x_{i}\sigma_{0}^{2}+\theta_{0}\sigma^{2}}{n\sigma_{0}^{2}+\sigma^{2}}\right)^{2}-\left(\frac{\sum_{i=1}^{n} x_{i}\sigma_{0}^{2}+\theta_{0}\sigma^{2}}{n\sigma_{0}^{2}+\sigma^{2}}\right)^{2}+\left(\frac{\sum_{i=1}^{n} x_{i}^{2}\sigma_{0}^{2}+\theta_{0}^{2}\sigma^{2}}{n\sigma_{0}^{2}+\sigma^{2}}\right)\right\}\right]$ $p(\mathbf{x}|\theta)\times p(\theta)=\frac{K}{\sigma_{0}\sigma^{n}(2\pi)^{\frac{n+1}{2}}}\exp\left[-\frac{1}{2\frac{\sigma^{2}\sigma_{0}^{2}}{\sigma^{2}+n\sigma_{0}^{2}}}\left\{\left(\theta-\frac{\sum_{i=1}^{n} x_{i}\sigma_{0}^{2}+\theta_{0}\sigma^{2}}{n\sigma_{0}^{2}+\sigma^{2}}\right)^{2}\right\}\right]$.

Where $K=\exp\left[-\frac{1}{2\frac{\sigma^{2}\sigma_{0}^{2}}{\sigma^{2}+n\sigma_{0}^{2}}}\left\{\left(\frac{\sum_{i=1}^{n} x_{i}^{2}\sigma_{0}^{2}+\theta_{0}^{2}\sigma^{2}}{n\sigma_{0}^{2}+\sigma^{2}}\right)-\left(\frac{\sum_{i=1}^{n} x_{i}\sigma_{0}^{2}+\theta_{0}\sigma^{2}}{n\sigma_{0}^{2}+\sigma^{2}}\right)^{2}\right\}\right]$

and

$\int_{-\infty}^{\infty} p(\mathbf{x}|\theta)\times p(\theta)d\theta$,

$\int_{-\infty}^{\infty} p(\mathbf{x}|\theta)\times p(\theta)d\theta=\frac{K}{\sigma_{0}\sigma^{n}(2\pi)^{\frac{n+1}{2}}}\int_{-\infty}^{\infty} \exp\left[-\frac{1}{2\frac{\sigma^{2}\sigma_{0}^{2}}{\sigma^{2}+n\sigma_{0}^{2}}}\left\{\left(\theta-\frac{\sum_{i=1}^{n} x_{i}\sigma_{0}^{2}+\theta_{0}\sigma^{2}}{n\sigma_{0}^{2}+\sigma^{2}}\right)^{2}\right\}\right]d\theta$,

after simplification, we have

$\int_{-\infty}^{\infty} p(\mathbf{x}|\mu)\times p(\theta)d\theta=\frac{K}{\sigma_{0}\sigma^{n}(2\pi)^{\frac{n+1}{2}}}\sqrt{\frac{\sigma^{2}\sigma_{0}^{2}}{\sigma^{2}+n\sigma_{0}^{2}}} \times\sqrt{2\pi}$,

thus the Pdistribution can be obtained as, $p(\theta|\mathbf{x})=\frac{1}{\sqrt{2\pi}\sqrt{\frac{\sigma^{2}\sigma_{0}^{2}}{\sigma^{2}+n\sigma_{0}^{2}}}}\exp\left[-\frac{1}{2}\left(\frac{\theta-\frac{\sum_{i=1}^{n} x_{i}\sigma_{0}^{2}+\theta_{0}\sigma^{2}}{n\sigma_{0}^{2}+\sigma^{2}}}{\sqrt{\frac{\sigma^{2}\sigma_{0}^{2}}{\sigma^{2}+\sigma_{0}^{2}}}}\right)^{2}\right]$, (B**1)**

$\theta|\mathbf{x}\sim N(\theta_{n},\sigma_{n}^{2})$, where $\theta_{n}=\frac{\sum_{i=1}^{n} \sigma_{0}^{2}x_{i}+\theta_{0}^{2}\sigma^{2}}{\sigma^{2}+n\sigma_{0}^{2}}$ and .

- 1. **Utilizing normal prior the posterior predictive distribution**

**The PP distribution can be derived as,**

$p(y|\theta)=\frac{1}{\sigma\sqrt{2\pi}}\exp[-\frac{1}{2}\left(\frac{y-\theta}{\sigma}\right)^{2}]$ and $p(\theta|\mathbf{x})$given in Eq. (C1)

Where$p(y|\mathbf{x})$ represents the predicted P distribution given as,

$p(y|\mathbf{x})=\int_{-\infty}^{\infty} \frac{1}{\sigma\sqrt{2\pi}}\exp[-\frac{1}{2}\left(\frac{y-\theta}{\sigma}\right)^{2}\times\frac{1}{\sqrt{2\pi}\sqrt{\frac{\sigma^{2}\sigma_{0}^{2}}{\sigma^{2}+n\sigma_{0}^{2}}}}\exp\left[-\frac{1}{2}\left(\frac{\theta-\frac{\sum_{i=1}^{n} x_{i}\sigma_{0}^{2}+\theta_{0}\sigma^{2}}{n\sigma_{0}^{2}+\sigma^{2}}}{\sqrt{\frac{\sigma^{2}\sigma_{0}^{2}}{\sigma^{2}+n\sigma_{0}^{2}}}}\right)^{2}\right]d\theta$,

for computational simplicity we consider, $\theta_{1}=\frac{\sum_{i=1}^{n} x_{i}\sigma_{0}^{2}+\theta_{0}\sigma^{2}}{n\sigma_{0}^{2}+\sigma^{2}}$and $\sigma_{1}=\sqrt{\frac{\sigma^{2}\sigma_{0}^{2}}{\sigma^{2}+n\sigma_{0}^{2}}}$,

after simplification, we have

$$p(y|\mathbf{x})=$$

$$\frac{1}{\sigma\sigma_{1}(2\pi)}\exp\left[-\frac{1}{2}\frac{\sigma^{2}+\sigma_{1}^{2}}{\sigma^{2}\sigma_{1}^{2}}\left\{\frac{(y^{2}\sigma_{1}^{2}+\theta_{1}^{2}\sigma^{2})(\sigma^{2}+\sigma_{1}^{2})-(y\sigma_{1}^{2}+\theta_{1}\sigma^{2})}{(\sigma^{2}+\sigma_{1}^{2})^{2}}\right\}\right]$$

$\times\int_{-\infty}^{\infty} \exp\left[-\frac{1}{2}\left\{\left(\frac{\theta-\frac{y\sigma_{1}^{2}+\theta_{1}\sigma^{2}}{\sigma_{1}^{2}+\sigma^{2}}}{\sqrt{\frac{\sigma^{2}\sigma_{1}^{2}}{\sigma^{2}+\sigma_{1}^{2}}}}\right)^{2}\right\}\right]d\theta$,

after further simplification, we have

$p(y|\mathbf{x})=\frac{1}{\sqrt{\sigma_{1}^{2}+\sigma^{2}}\sqrt{2\pi}}\exp[-\frac{1}{2}\left(\frac{y-\theta_{1}}{\sqrt{\sigma_{1}^{2}+\sigma^{2}}}\right)^{2}$. **(B2)**

Thus $y|\mathbf{x}$ is normally distributed with mean $\frac{\sum_{i=1}^{n} x_{i}\sigma_{0}^{2}+\theta_{0}\sigma^{2}}{n\sigma_{0}^{2}+\sigma^{2}}$ and variance$\sigma^{2}+\frac{\sigma^{2}\sigma_{0}^{2}}{\sigma^{2}+n\sigma_{0}^{2}}$.

Let $y_{1},y_{2,}y_{3},...,y_{k}$ represent a future observation of size *k.* In this scenario, $Y|X$ follows a normal distribution with a mean of $\frac{\sum_{i=1}^{n} x_{i}\sigma_{0}^{2}+\theta_{0}\sigma^{2}}{n\sigma_{0}^{2}+\sigma^{2}}$ and a variance of $\frac{\sigma^{2}}{k}+\frac{\sigma^{2}\sigma_{0}^{2}}{\sigma^{2}+n\sigma_{0}^{2}}$.

# Appendix-C

- 1. **under SELF the Bayes and predicted Bayes estimators**

To demonstrate the validity of expression A1, we proceed by utilizing expression B1.

$\theta_{SELF}=E_{\theta|\mathbf{x}}(\theta)=\int_{-\infty}^{\infty} \theta p(\theta|\mathbf{x})d\theta$,

$\hat{\theta}_{SELF}=E_{\theta|\mathbf{x}}(\theta)=\int_{-\infty}^{\infty} \theta\frac{1}{\sqrt{2\pi}\sqrt{\frac{\sigma^{2}\sigma_{0}^{2}}{\sigma^{2}+n\sigma_{0}^{2}}}}\exp\left[-\frac{1}{2}\left(\frac{\theta-\frac{\sum_{i=1}^{n} x_{i}\sigma_{0}^{2}+\theta_{0}\sigma^{2}}{n\sigma_{0}^{2}+\sigma^{2}}}{\sqrt{\frac{\sigma^{2}\sigma_{0}^{2}}{\sigma^{2}+\sigma_{0}^{2}}}}\right)^{2}\right] d\theta$,

after Simplification, we have

$\hat{\theta}_{SELF}=E_{\theta|\mathbf{x}}(\theta)=\frac{\sum_{i=1}^{n} x_{i}\sigma_{0}^{2}+\theta_{0}\sigma^{2}}{n\sigma_{0}^{2}+\sigma^{2}}$,

$\hat{\theta}_{SELF}=\frac{n\bar{x}\sigma_{0}^{2}+\theta_{0}\sigma^{2}}{n\sigma_{0}^{2}+\sigma^{2}}$.

The equation representing the Bayes predicted estimator under SELF is provided as Eq. (A2).

We compute using the Eq. (B2) as,

$$\hat{\theta}_{SELF1}=E_{y|\mathbf{x}}(y)=\int_{-\infty}^{\infty} \theta p(y|\mathbf{x}) dy$$

$\hat{\theta}_{SELF1}=E_{y|\mathbf{x}}(y)=\int_{-\infty}^{\infty} \theta\frac{1}{\sqrt{\sigma_{1}^{2}+\sigma^{2}}\sqrt{2\pi}}\exp[-\frac{1}{2}\left(\frac{y-\theta_{1}}{\sqrt{\sigma_{1}^{2}+\sigma^{2}}}\right)^{2}dy$,

after simplification, we have

$\hat{\theta}_{SELF1}=E_{y|\mathbf{x}}(y)=\theta_{1}\Rightarrow\frac{\sum_{i=1}^{n} x_{i}\sigma_{0}^{2}+\theta_{0}\sigma^{2}}{n\sigma_{0}^{2}+\sigma^{2}}$,

or

$\hat{\theta}_{SELF1}=\frac{n\bar{x}\sigma_{0}^{2}+\theta_{0}\sigma^{2}}{n\sigma_{0}^{2}+\sigma^{2}}$.

Given that the samples are obtained through various RSS schemes, the calculation involves determining the mean and variance of the Bayes estimator and Bayes predictor estimator under the SELF. These calculations are derived in detail in Appendix-C, specifically considering the SELF as the chosen LF.

The Bayes estimator under SELF under RSS given as

,

the mean and variance of the derived as

, where $E\left(\overline{x}_{(RSS)}\right)=\mu$.

And

,

, where $V\left(\overline{x}_{(RSS)}\right)=\frac{\delta^{2}}{m}-\frac{1}{m^{2}}\sum_{i=1}^{m} \left(\mu_{\left(i\right)}-\mu\right)$.

The *Bayes* estimator under SELF under MRSS given as

,

, where $E\left(\overline{x}_{(MRSS)}\right)=\mu$.

And

,

,

where $V\left(\overline{x}_{(MRSS)}\right)$ in case odd sample size

$V\left(\overline{x}_{(MRSS)}\right)=\frac{1}{m}\left(\delta_{\left(\frac{m+1}{2}\right)}^{2}\right)$,

and when the sample is even than the $V\left(\overline{x}_{(MRSS)}\right)$ is given by

$V\left(\overline{x}_{(MRSS)}\right)=\frac{1}{m}\left(\delta_{\left(\frac{m}{2}\right)}^{2}+\delta_{\left(\frac{m+2}{2}\right)}^{2}\right)$.

The *Bayes* estimator under SELF under ERSS given as

,

the mean and variance of the is derived as

, where $E\left(\overline{x}_{(ERSS)}\right)=\mu$.

And

,

,

where $V\left(\overline{x}_{(ERSS)}\right)$ in case odd sample size

$\mathrm{var}\left(\overline{x}_{\left(ERSS\right)O}\right)=\frac{1}{2m^{2}}\left(\delta_{\left(1\right)}^{2}+\delta_{\left(m\right)}^{2}\right)+\frac{1}{m^{2}}\left(\delta_{\left(\frac{m+1}{2}\right)}^{2}\right)$,

and when the sample size is even than $V\left(\overline{x}_{(ERSS)}\right)$ is given as

$\mathrm{var}\left(\overline{x}_{\left(ERSS\right)e}\right)=\frac{1}{2m}\left(\delta_{\left(1\right)}^{2}+\delta_{\left(m\right)}^{2}\right)$.

The same procedure is adopted for LLF.

# Appendix-D

# TABLE D1: Comparison of variances under different sampling schemes

| $n$ | **SRS** |  | **RSS** |  | **MRSS** |  | **ERSS** |  |
| --- | --- | --- | --- | --- | --- | --- | --- | --- |
|  |  |  |  |  |  |  |  |  |
| 3 | 0.185 | 0.181 | 0.099 | 0.096 | 0.084 | 0.081 | 0.097 | 0.098 |
| 4 | 0.158 | 0.153 | 0.067 | 0.066 | 0.058 | 0.055 | 0.063 | 0.078 |
| 5 | 0.138 | 0.130 | 0.049 | 0.050 | 0.039 | 0.035 | 0.041 | 0.057 |
| 6 | 0.123 | 0.121 | 0.037 | 0.036 | 0.030 | 0.029 | 0.036 | 0.050 |

**Appendix E**

**List of Abbreviations and Notations**

| CC | Control Chart |
| --- | --- |
| SQC | Statistical Quality Control |
| SPC | Statistical Process Control |
| EWMA | Exponentially Weighted Moving Average |
| CUSUM | Cumulative Sum |
| AEWMA | Adaptive Exponentially Weighted Moving Average |
| LF | Loss Function |
| SELF | Squared Error Loss Function |
| P | Posterior |
| PP | Posterior Predictive |
| LLF | Linex Loss Function |
| ARL | Average Run Length |
| SDRL | Standard Deviation of Run Length |
| *ARLo* | ARL for in-control process |
| *ARL*1 | ARL for out-of-control process |
| *UCL* | Upper control limit |
| *CL* | Center line |
| *LCL* | Lower control limit |
| SRS | Simple Random Sampling |
| RSS | Ranked Set Sampling |
| MRSS | Median Ranked Set Sampling |
| ERSS | Extreme Ranked Set Sampling |
| $\theta$ | Mean of normal distribution |
| $\delta^{2}$ | Variance of normal distribution |
| $\theta_{0}$ | Mean of prior distribution |
| $\delta_{0}^{2}$ | Variance of prior distribution |
| $\theta_{n}$ | Mean of posterior distribution |
| $\delta_{n}^{2}$ | Variance of posterior distribution |
| $\bar{x}$ | Mean of study variable obtain form SRS |
| $\overline{x}_{(RSS)}$ | Mean of study variable obtain form RSS |
| $\overline{x}_{(MRSS)}$ | Mean of the study variable obtain from MRSS |
| $\overline{x}_{(ERSS)}$ | Mean of the study variable obtain from ERSS |
|  | Bayes estimator under SELF |
|  | Bayes estimator under LLF |
| $\delta_{1}^{2}$ | Variance for posterior predictive distribution |
| $\partial$ | Shift |

Bottom of Form
